# Tumour SUVmean and volume relates to its radiation absorbed dose

**DOI:** 10.1186/s41824-026-00319-2

**Published:** 2026-08-24

**Authors:** Yung Hsiang Kao

**Affiliations:** Department of Nuclear Medicine, The Royal Melbourne Hospital, 300 Grattan Street, Parkville, VIC 3050 Australia

Recent interest in PET metrics such as the SUVmean and tumour volume requires re-direction from a radiobiologic perspective. For example, Kiani et al. recently performed a comprehensive meta-analysis on the prognostic value of PSMA PET biomarkers for response to lutetium-177 PSMA therapy in metastatic castration resistant prostate cancer (Kiani et al. 2026). They found SUVmean and tumor volume to be significant prognostic biomarkers. For overall survival, Kiani et al. found SUVmean to have a hazard ratio of 0.93 (95% confidence interval 0.90–0.96) and tumor volume to have a hazard ratio of 1.37 (95% confidence interval 1.27–1.48) (Kiani et al. 2026). This means that better survival is directly related to higher SUVmean, but inversely related to tumour volume. In notation form:

$$\:Survival\propto\:SUVmean$$

Relationship 1

$$\:Survival\propto\:\frac{1}{Tumor\:volume}\:$$

Relationship 2

Since the SUVmean is simply a ratio that describes the relative biodistribution of systemically injected radioactivity into tumor, Relationship 1 may be re-expressed as:

$$\:Survival\propto\:Tumor\:radiation\:energy$$

And since tumor volume is directly proportional to its mass, combining Relationships 1 and 2 obtains:

$$\:Survival\propto\:\frac{Tumor\:radiation\:energy}{Tumor\:mass}$$

By definition, therefore:

$$\:Survival\propto\:Tumor\:radiation\:absorbed\:dose\:\left(Gy\right)$$

From this perspective, we can therefore say that Kiani et al. has shown the key prognostic metric in systemic radiopharmaceutical therapy to be none other than the radiation absorbed dose, expressed in gray (Gy). This radiobiologic perspective empowers the enlightened reader to see through today’s tangled web of PET metrics as imperfect surrogates of the radiation absorbed dose. Henceforth, work on systemic radiopharmaceutical therapy should focus on the personalized radiation absorbed dose delivered in fractions, not ‘cycles’ of empirically prescribed radioactivity (Bq). An example of a clinically validated and implemented solution for rapid predictive dosimetry in systemic radiopharmaceutical therapy is now freely available (Kao et al. 2024, Kao 2025, Kao et al. 2026).

## Data Availability

The dataset supporting the conclusions of this article is included within the article.

## Abbreviations

PET: Positron emission tomography
SUV: Standardised uptake value
PSMA: Prostate specific membrane antigen
